# Progress of Epidemics in Forty-One Towns and Districts

**Published:** 1858-01

**Authors:** 


### PROGRESS OF EPIDEMICS.

### LOCAL REPORTS OF EPIDEMIC AND ENDEMIC DISEASES

*During the Months of September, October, and November,* 1857.

| Place. | County. | Lat. | Long. | Observer. |
|---|---|---|---|---|
| St. Mary, Scilly | Cornwall - | 49.50 N. | 6.24 W. | J. G. Moyle, Esq. |
| Teignmouth - | Devonshire - | 50.32 N. | 3.29 W. | W. C. Lake, Esq. |
| Odiham - - | Hampshire - | 51. 8 N. | 1. 3 W. | J. McIntyre, M.D. |
| Canterbury - | Kent - - | 51.17 N. | 1. 4 E. | G. Rigden, Esq. |
| Chatham - | Kent - - | 51.21 N. | 0.14 E. | F. J. Brown, M.D. |
| Wandsworth - | Surrey - - | 51.28 N. | 0. 7 W. | G. E. Nicholas, Esq. |
| Putney - - | Surrey - - | 51.28 N. | 0. 8 W. | R. H. Whiteman, Esq. |
| Up. Holloway | Middlesex - | 51.32 N. | 0.03 E. | W. B. Kesteven, Esq. |
| Swansea - | Glamorgansh. | 51.38 N. | 3.50 W. | W. H. Michael, Esq. |
| Colchester - | Essex - - | 51.54 N. | 0.52 E. | Henry Laver, Esq. |
| Aspley Guise - | Bedfordshire - | 52. 1 N. | 0.37 W | J. Williams, M.D. |
| Saffron Walden | Essex - - | 52. 3 N. | 0.12 E. | T. Spurgin, Esq.<br>H. Stear, Esq. |
| Bedford - - | Bedfordshire - | 52. 8 N. | 0.51 W. | T. H. Barker, M.D. |
| Newpt. Pagnell | Buckinghams. | 52.10 N. | 0.42 W. | G. O. Rogers, Esq. |
| Sharnbrook - | Bedfordshire - | 52.12 N. | 0.40 W. | R. S. Stedman, Esq. |
| Wellingbro' - | Northamptons. | 52.20 N. | 0.40 W. | B. Dulley, Esq. |
| Beccles - - | Suffolk - - | 52.25 N. | 1.48 E. | W. E. Crowfoot, Esq. |
| Thetford - | Norfolk - - | 52.26 N. | 0.45 E. | H. W. Bailey, Esq. |
| Stourbridge - | Worcestersh. - | 52.28 N. | 2.32 W. | A. Freer, Esq. |
| Dudley - - | Staffordshire - | 52.30 N. | 2.10 W. | J. H. Houghton, Esq. |
| Barrowden - | Rutland - | 52.34 N. | 0.38 W. | H. J. Swann, Esq. |
| Wisbeach - | Cambridgesh. | 52.39 N. | 0. 5 E. | W. H. Hole, Esq. |
| East Dereham | Norfolk - - | 52.40 N. | 0.57 E. | J. Vincent, M.D. |
| Pontesbury - | Shropshire - | 52.43 N. | 2.50 W. | Wm. Eddowes, Esq. |
| Nottingham - | Nottinghamsh. | 52.50 N. | 1.10 W. | T. Robertson, M.D. |
| Burton-on-Trt. | Staffordshire - | 52.53 N. | 1.53 W. | S. Thomson, M.D. |
| Oswestry - | Shropshire - | 52.53 N. | 2.59 W. | P. Cartwright, Esq. |
| Wrexham - | Denbighshire - | 53. 2 N. | 3. 1 W. | E. Williams, M.D. |
| Hawarden - | Flintshire - | 53.11 N. | 3. 2 W. | T. Moffat, M.D. |
| Lincoln - | Lincolnshire - | 53.12 N. | 0. 5 W. | S. Lowe, Esq. |
| Alford - - | Lincolnshire - | 53.15 N. | 0. 6 E. | R. U. West, M.D. |
| Gainsborough | Lincolnshire - | 53.23 N. | 0.47 W. | D. Mackinder, M.D. |
| Liverpool - | Lancashire - | 53.24 N. | 2.59 W. | Thos. Bickerton, Esq. |
| Warrington - | Lancashire - | 53.25 N. | 2.32 W. | C. N. Spinks, Esq. |
| Wigan - - | Lancashire - | 53.32 N. | 2.33 W. | Jephtha Hussey, Esq. |
| Bolton - - | Lancashire - | 53.35 N. | 2.19 W. | W. H. Pendlebury, Esq. |
| Bramley - | Yorkshire - | 53.49 N. | 1.38 W. | J. N. Radcliffe, Esq. |
| York - - | Yorkshire - | 53.58 N. | 1. 3 W. | W. Procter, Esq. |
| Wst. Auckland | Durham - | 54.45 N. | 1.40 W. | G. Todd, Esq. |
| Rothbury - | Northumberld. | 55.25 N. | 1.50 W. | E. C. Summers, Esq. |
| Lerwick - | Shetland Isles | 60. 9 N. | 1. 8 W. | G. W. Spence, M.D. |

### QUARTERLY STATEMENT—No. XII.

[*The dates denote that the disease appeared in the weeks then ending.*]

SCARLET FEVER.

Canterbury—Sept. 11, all Oct. and Nov.
Chatham—September 18
Putney—October 16-30, all Nov.
Upper Holloway—October 16, 30
Bedford—All November
Beccles—All Sept., Oct. 2-16, all Nov.
Thetford—November 6-13, 27
Stourbridge—Sept. 11-18, Oct. 2, 16, 30
Dudley—Sept. 18-25, Oct. 2, 16, Nov. 13-27
Wisbeach—November 20-27
Wrexham—All November

Hawarden—November 13
Lincoln—Oct. 23-30, all November
Gainsborough—Every wk. except Nov. [20
Liverpool—Every week
Wigan—October 9, November 6, 27
Bolton-le-Moors—Sept. 4-18, Oct. 9-30, all November
York—Sept. 11, 25, October 2, 16-30
West Auckland—Every week

MEASLES.

Scilly Isles—Sept. 11, October 2-16
Canterbury—All November
Chatham—Sept. 25, Nov. 20-27
Wandsworth—November 20
Colchester—September 25
Saffron Walden—September 11-18
Beccles—All November
Thetford—September 4-18
Dudley—Sept. 4, 25, October 16
Barrowden—Oct. 9-30, all November
East Dereham—All November
Lincoln—October 2-16
Liverpool—Every week
Gainsborough—Sept. 25, October 2
Bolton-le-Moors—Sept. 18 25, Oct. 2, 23-30, November 6-13
Bramley—November 20

SMALL POX.

Bedford—November 20-27
Stourbridge—September 4-18
Dudley—October 30, November 13
Wisbeach—October 23-30, all Nov.
Pontesbury—November 13-27
Oswestry—October 2
Wrexham—November 27
Hawarden—October 23-30
Liverpool—Every week
Wigan—Sept. 26, October 16, Nov. 6
Bolton-le-Moors—Sept. 4-11, October 23-30, November 6
York—Sept. 4-18, all Oct., Nov. 6-20

HOOPING COUGH.

Chatham—Sept. 11-25, October 2, 16, November 13-27
Upper Holloway—All Sept., Nov. 13-[27
Colchester—All October & November
Saffron Walden—Sept. 11, Oct. 23
Bedford—November 6-13
Sharnbrook—November 13-27
Wellingborough—November 27
Dudley—October 9, 30, Nov. 6, 27
Wisbeach—All November
East Dereham—All October and Nov.
Pontesbury—All Sept. & Oct., Nov. 6
Burton-on-Trent—All September
Oswestry—Every week
Wrexham—October 16-30, Nov. 6-13
Lincoln—November 13
Alford—October 9-16
Gainsborough—September 18
Liverpool—Every week
Wigan—September 25, November 13
Bolton le-Moors—Sept. 11-18, Oct. 2-[16, 30, all Nov.
York—Oct. 2, 30

CROUP.

Chatham—Sept. 4, Oct. 2, Nov. 20-27
Putney—September 25, October 2
Aspley Guise—November 20-27
Saffron Walden—October 30
Newport Pagnell—November 6
Thetford—November 6-13
Stourbridge—November 13-27
Wisbeach—November 20-27
Pontesbury—October 23
Burton-on-Trent—November 13
Oswestry—October 2-9, Nov. 13-27
Lincoln—November 13-27
Alford—September 25
Liverpool—Sept. 18-25, Oct. 2-9, 30, [Nov. 13-27
Bolton-le-Moors—Sept. 25, Oct. 2, 30, [Nov. 6-13
Bramley—November 20

CATARRH.

Scilly Isles—All November
Teignmouth—All Sept., Oct. 2, Nov. 6-[13, 27
Odiham—October 16-30, Nov. 6-20
Chatham—Sept. 4, Oct. 16-30, all Nov.
Wandsworth—All Sept., October 2-9, 23-30, November 13-27
Putney—September 11-25, Oct. 2-9
Upper Holloway—October 30, Nov. 6
Aspley Guise—All November
Saffron Walden—Sept. 25, Nov. 13-27
Bedford—November 27
Newport Pagnell—All November
Sharnbrook—Every week
Wellingborough—November 13 27
Beccles—Sept. 11-18, October 9-30
Thetford—Sept. 4-11, 25, October 9-16, 30, all November
Stourbridge—Sept. 4-18, Oct. 9-30, all [Nov.
Dudley—October 30
Wisbeach—All October & November
Burton-on-Trent—November 6-20
Oswestry—November 20-27
Wrexham—October 30, all November
Gainsborough—Sept. 25, all Oct. & Nov.
Liverpool—Every week
Bolton-le-Moors—Sept. 11-18, Oct. 9-[30, all Nov.
Bramley—All Nov.
Rothbury—November 20-27
Lerwick—Sept. 4, Oct. 23, Nov. 13-27

INFLUENZA.

Teignmouth—Sept. 11-18, Nov. 20-27
Odiham—October 30, Nov. 6-13
Chatham—Sept. 11, 25, Oct. 16-30, all [Nov.
Wandsworth—Nov. 13 27
Putney—October 16-30, all Nov.
Upper Holloway—November 6, 20-27
Colchester—November 13 27
Aspley Guise—November 20-27

Saffron Walden—Sept. 18-25, Oct. 2,
Bedford—Nov. 13-27 [Nov. 13-27
Newport Pagnell—Sept. 4-11, 25, Oct.
Wellingborough—All Nov. [2, all Nov.
Thetford—Sept. 11, 25, Oct. 2-9, 23, all
Stourbridge—Nov. 20-27 [Nov.
Pontesbury—November 13, 27
Wrexham—All November
Hawarden—October 16-23
Lincoln—October 30, all November
Liverpool—Every week
Wigan—Oct. 16-23, Nov. 6, 20-27
Bolton-le-Moors—Every week
Bramley—November 20-27
York—Sept. 25, all Oct., Nov. 6-20
Lerwick—November 27

ERYSIPELAS.

Odiham—November 13
Canterbury—Sept. 25 [Nov. 13-20
Chatham—Sept. 11, 25, Oct. 2, 16, 30,
Wandsworth—Sept. 18, Oct. 23-30,
Putney—November 13 [Nov. 6
Saffron Walden—Sept. 25, Nov. 13
Bedford—September 4 11
Newport Pagnell—Sept. 4-11, Oct. 16-
Sharnbrook—Oct. 9-16 [30, Nov. 6
Wellingborough—Sept. 25, Oct. 2-9, November 20-27
Beccles—September 4, Nov. 13-20
Thetford—Sept. 4, 18, October 16
Stourbridge—October 30, Nov. 20
Dudley—Sept. 4, Oct. 2, Nov. 27
Wisbeach—November 20-27
Pontesbury—September 4-18
Burton-on-Trent—October 9
Oswestry—All Sept., Oct. 2, Nov. 20-27
Lincoln—All November
Alford—October 9
Gainsborough—Sept. 4, Oct. 9-16, Nov.
Liverpool—Every week [6-13
Bolton-le-Moors—Sept. 11, Oct. 9-16,
Bramley—Nov. 27 [30, Nov. 6-13
Lerwick—September 4

CHOLERA (ENGLISH).

Chatham—September 4, 18
Newport Pagnell—Sept. 18-25, Oct. 9-
Wellingborough—Sept. 4-18 [23
Beccles—September 18
Thetford—Sept. 18, Oct. 9, 30, Nov. 6
Stourbridge—All Sept., Nov. 27
Pontesbury—September 4, 18
Wrexham—September 11-25
Alford—September 18 25
Liverpool—All Sept., Oct. 2, 16
Bolton le-Moors—Sept. 4-18, Oct. 2-9
Bramley—September 25, October 2

AGUE.

Canterbury—Sept. 11-18, all Oct. & Nov.
Chatham—Every week
Colchester—Every week
Wisbeach—Every week
Alford—October 16-23
Liverpool—September 11, 25

REMITTENT FEVER.

Teignmouth—Sept. 11-25, Nov. 6, 20-27
Odiham—Oct. 9, 23 30, Nov. 6, 20
Putney—Oct. 16-30, Nov. 6-13
Upper Holloway—Sept. 18-25
Saffron Walden—Oct. 2-16, Nov. 13-27
Newport Pagnell—September 18-25
Beccles—Sept. 4, Oct. 23-30, Nov. 6
Thetford—Sept. 11, 25, Oct. 16
Dudley—September 2
Barrowden—Sept. 11-18, Oct. 30
Burton-on-Trent—September 18-25
Oswestry—All October and Nov.
Alford—Sep. 11-25, Oct. 9 16, Nov. 20 27
Gainsborough—Sept. 18-25, Oct. 2-9
Liverpool—Every week
Wigan—Oct. 16-23, Nov. 6
Bolton-le-Moors—Sept. 18-25, Oct. 2-9, 30, all November
York—Sept. 11, 25, Oct. 30, Nov. 6

DIARRHŒA.

Scilly Isles—All Sept., Oct. 2-16, all Nov.
Teignmouth—All Sept., Oct. 2-9, Nov.
Odiham—October 2-16 [6, 20
Canterbury—Every week
Chatham—All Sept. & Oct., Nov. 6-13
Wandsworth—All Sept., Oct. 2-16, 30, November 6
Putney—All Sept., Oct. 2-16, Nov. 13-20
Upper Holloway—All September
Colchester—Every week
Aspley Guise—All Sept., Oct. 2-16
Saffron Walden—Every week
Bedford—Sept. 4-11, 25, Oct. 16-23, November 6-13
Newport Pagnell—Every week except
Sharnbrook—Every week [last
Wellingborough—All Sept., Oct. 2-16
Beccles—All September
Thetford—All Sept., Oct. 2-9, 23 30, Nov. 6-13, 27 [20-27
Stourbridge—All Sept., Oct. 9-23, Nov.
Dudley—All Sept., Oct. 2, 16-30, Nov. 6, 20-27 [Nov. 13
Barrowden—Sept. 11 25, Oct. 9-30,
Wisbeach—All Sept., Oct. 2-9
East Dereham—All Sept., Oct. 2
Pontesbury—Sept. 11-25, Oct. 2-23
Burton-on-Trent—Sept. 11-25, Oct. 9,
Oswestry—Every week [23-30
Wrexham—All Sept, Oct. 2-16
Hawarden—All October, Nov. 6-20
Lincoln—October 16-30, Nov. 6
Alford—All September and October
Gainsborough—All Sept. & Oct., Nov. 6
Liverpool—Every week
Wigan—Sept. 4-18, Oct. 16, 30

Bolton-le-Moors—All Sept., Oct. 2-16, 30, November 6
Bramley—Sept. 4, 18-25, Oct. 2-16, 30
York—Oct. 9, 23-30, Nov. 13-20 [6
Rothbury—Sept. 11, 25, Oct. 16-30, Nov.
Lerwick—Sept. 4, 25, Oct. 16-30, Nov. 6-20

DYSENTERY.

Odiham—November 20-27
Chatham—All Sept., October 2-16, 30, November 13 [20
Wandsworth—Sept. 4, 18, Oct. 2, Nov.
Saffron Walden—Sept. 11, Oct. 2
Newport Pagnell—Sept. 4, Nov. 20
Sharnbrook—Sept 4, Oct. 30
Wellingborough—All Sept., Oct. 2
Stourbridge—September 4-11
Wrexham—All September, Oct. 2
Lincoln—October 16-23
Alford—All September
Gainsborough—All Sept., Oct. 2-9
Liverpool—Every week
Wigan—Sept. 4, 18, Oct. 16
Bolton-le-Moors—All Sept., Oct. 2-9, November 6-20
Bramley—All Sept., Oct. 2, 16, 30, Nov.
Lerwick—September 25 [6, 27

TYPHUS.

Canterbury—Every week
Chatham—Every week
Wandsworth—Sept. 4, Nov. 13
Putney—October 30
Upper Holloway—Sept. 25, Oct. 2
Saffron Walden—Every week
Newport Pagnell—Sept. 18-25, all Oct.
Sharnbrook—Oct. 9-16, 30 [& Nov.
Wellingborough—Every week
Beccles—All Sept., Oct. 23-30, Nov. 6-13
Thetford—Sept. 4-11, 25, Oct. 9, 23-30,
Dudley—Every week [Nov. 13-27
East Dereham—All November
Pontesbury—November 13-27
Oswestry—Sept. 18, Oct. 9
Wrexham—September 4-18
Lincoln—Oct. 9-30, all November
Alford—September 4, November 6
Gainsborough—All Oct. and Nov.
Liverpool—Every week
Wigan—September 4, October 16
Bolton-le-Moors—Sept. 4-11, Oct. 2-9,
York—Oct. 9, 22 [23-30, all Nov.
West Auckland—All Oct. and Nov.
Rothbury—Oct. 2-9, Nov. 20

TYPHOID FEVER.

Colchester—Every week
Stourbridge—September 4-11
Lerwick—November 6

PUERPERAL FEVER.

Thetford—October 23
Alford—September 18
Bolton-le-Moors—Sept. 18-25, Oct. 23

CARBUNCLE.

Teignmouth—November 20
Odiham—Sept. 25, Nov. 20
Canterbury—All September
Chatham—October 16, Nov. 6
Colchester—September 18
Saffron Walden—Sept. 11, Oct. 2-9, 30, November 13-27
Sharnbrook—Sept. 4, all Nov.
Thetford—November 13
Stourbridge—November 6-13
Oswestry—October 2-16, Nov. 27
Alford—Sept. 4-11, Oct. 30
Liverpool—Sept. 11, 25, Oct. 2, 16-23, November 6, 20-27
Bolton-le-Moors—Sept. 11-18, Oct. 23
Rothbury—October 2

MUMPS.

Lerwick—October 30, Nov. 20

DIPHTHERITE.

Canterbury—Every week

---

### ADDITIONAL OBSERVATIONS.

*Scilly Isles.*—Mr. Moyle observes: "We had one case of measles in September, and three in the first half of October, all mild in character. The case in September was imported into the island from Newlyn, a fishing town close to Penzance, where measles were rife. The diarrhœa in October and November attacked more particularly children, and especially infants at the breast. It was attended, in many instances, with choleraic vomiting. Several of the infants were almost in a state of collapse, cold and blue: they all recovered, however, with stimulating remedies; one infant at the breast taking twenty drops of brandy at a dose. Chalk mixture, with a small proportion of opiate, stilled the diarrhœa. The cases of catarrh were numerous, but uncomplicated, and only requiring common domestic remedies. The weather during September was very fine and warm, the mean temperature for the month being 68° Fahrenheit: the prevailing winds were from the east, and the rain gauged 2.69 in. October was a very wet month, the rain gauged amounting to the large quantity (for Scilly) of 4.60 inches. Several gales of wind occurred, and the whole month was cold and uncomfortable. The mean temperature was 52° Fahr., the prevailing winds north-east and south-east. November to the 27th has been fine. The rain gauged has been 1.27 inch. The weather has been mostly clear, and the prevailing winds from the east and the south. The mean temperature of the month has been 58° Fahr. The deaths for the quarter have been six: one from traumatic tetanus; one from phthisis in a woman; one from marasma from aphthous ulceration of the mouth and fauces in a child; one from softening of the brain in a woman; one from laryngismus stridulus (a child); one woman from uterine hæmorrhage. The population of the islands is 2,600."

*Teignmouth.*—Mr. Lake writes: "September was, on the whole, a fine month; the temperature decreasing, but still above the average, the difference between the degree of humidity of this month and the last being greater than ordinary; the fall of rain 1.112 in.; about the average. October was a very rainy month, the temperature being still above the average. Barometric pressure during this month was low. On the 8th it fell lower than I had before registered it (28.723). The total amount of rain that had fallen in the four months of June, July, August, and September, had been 3.608 inches. In October alone there fell 4.666 inches. November was at first mild and damp; during the middle of the month cold, with N. and N.E. winds; then for a few days mild and damp, and during its close again cold and bleak. From October 7th to 21st barometric pressure was high. On the 12th the glass reached a higher point than I had before noticed it at, viz. 30.671. 1.846 inches of rain fell during this month. The mean degree of ozone for the three months of the quarter was as follows:—September, 5.6; October, 4.8; November, 3.1. Diarrhœa had not appeared in force till the latter part of August. It prevailed increasingly through September, subsiding towards the end of October. Tonsillitis was common during the latter part of October and in November. Some cases came under my notice, amongst children, of a diphtheritic character, but in which the local affection was not so formidable as the constitutional. The blood-making powers of the system were apparently much compromised; the little patient, after the subsidence of the throat affection, either sinking through a general failure of the powers of life (without anything like typhoid symptoms), or left in a state in which he is liable to be carried off by any prevalent disorder; or, during convalescence, continuing unusually weak and anæmic."

*Chatham.*—Dr. Brown writes: "Typhoid fever has continued to prevail. The complications have been both cephalic and abdominal. Intestinal hæmorrhage has proved fatal in many cases. The head affection has presented the following characteristics :—Intense tinnitus, vigilance, flushed face, and, in some cases, screaming and delirium. The symptoms have, in almost all cases, yielded to a few leeches, small blisters, the adminstration of iodide of potassium, and the inunction of strong mercurial ointment over the forehead. Diphtheritis was observed in the week ending 23rd October, and in the same week cases of cholerine. The influenza, that has prevailed during the last seven weeks, has been marked by severe but short-continued symptoms. It has put on the character of suffocative catarrh, which has suddenly disappeared in less than four—in even two days. A girl, aged 25 years, died in two days of this affection in the week ending 27th November. Very many persons were prostrated on one day, and walking about a few days afterwards. A convulsive cough,—a quasi-hooping-cough,—now succeeds to the influenza, and affects adults as well as children."

*Wandsworth.*—Mr. Nicholas says: "The past quarter has been characterised by an almost entire absence of the exanthemata. Diarrhœa prevailed extensively at the commencement, and influenza, with much lung disease, at the latter part of the above period. During the continuance of the last-named epidemic, all disease was attended by a very marked depression of the vital powers.

*Putney.*—Mr. Whiteman writes: "Diarrhœa, of which several cases assumed a severe choleraic form, was the prevailing epidemic during September and the early part of October. Scarlatina, although very prevalent during October and November, was comparatively light and yielding, resulting in death in no one instance. Catarrhal affections, and that severe form of catarrhal fever, known as influenza, have been unusually prevalent throughout the entire quarter. The mortality of the district has been very considerably below the average of corresponding seasons of many years past. A good criterion of the state of the public health of a district is always to be found in the records of disease and mortality amongst the poor of such district. My last quarterly summary, as returned to the Board of Guardians, was of a most satisfactory character. Out of about two hundred cases of disease, of greater or less severity, there occurred but two deaths in this parish amongst the pauper class, in the thirteen weeks referred to, viz., a woman, aged 99, from natural decay, and a child, aged 7, who succumbed to a very severe burn. Not one poor person, therefore, can be said to have died during the quarter from actual disease."

*Colchester.*—Mr. Laver says: "During this quarter vegetation has generally been healthy, excepting potatoes, which are much diseased in many parts; apples appear to be diseased in a similar manner to potatoes; there have also been a few isolated cases of pleuropneumonia amongst cattle. Typhoid has been very prevalent, particularly in low and marshy districts with a bad water supply. The large number of cases of tonsillitis I have seen have appeared to be infectious, whole families suffering. I have not seen or heard of any cases of diphtherite. The worst cases of typhoid I have attended have occurred in patients drinking water from two shallow wells, the water of which is full of infusoria."

*Saffron Walden.*—Mr. Spurgin says: "The quarter has been unusually healthy, and, with the exception of diarrhœa, there has been no general disease. Vegetation has been unusually late, from the extreme mildness of the weather and absence of frost, which has prolonged the growth of the vegetable kingdom."

Mr. Stear says: "Diarrhœa still continues, but the cases are neither so severe or numerous as they were. Pulmonary affections have been frequent during the last month of the quarter. Fever cases are more frequent at the present time than they have been at any time before during the three years I have held the district, though the number of sick poor is below the average. Grass, etc., has grown remarkably during the past autumn. There has neither been any frosty weather nor fall of snow at present."

*Bedford.*—Dr. Barker reports that fever prevailed in this town and neighbourhood during the entire quarter.

*Sharnbrook.*—Mr. Stedman says: "Diarrhœa has been prevalent during the whole of the quarter. Of typhus, four cases came under my notice, two of which were fatal. In these two, epistaxis was of almost daily occurrence, and also most profuse perspiration. In a village about six miles from here, fever has prevailed to a great extent. The population, according to the last census, is 845; and I am informed by Mr. Purday, the medical officer of the district, that for some weeks during the last two months, he has been returning as many as one hundred and twenty cases to the Board of Guardians weekly, exclusive of cases occurring in private practice. This village, Bozeat, is notorious for its deficient drainage, and has a wide open ditch traversing it, which receives all the filth and drainage of the village. Several very heavy falls of rain have occurred during the quarter. Up to this date (November 30th) there has been no frost. Many half-hardy plants,—such as the scarlet geranium, the heliotrope, nasturtium, fuschia, French beans, and others, which are generally the first to die from cold, are still green, and in some cases bearing flowers."

*Wellingborough.*—Mr. Dulley writes: "Although the cases of fever are entered under the head of typhus, they have been, with one exception, typhoid cases, and seem to be of an epidemic character, visiting alike the ill-drained and the best drained part of the town, in which have been recently laid down new sewerage drains and pipes, very badly done, so much so that the pipes are frequently useless in carrying off the filth of the neighbourhood, and thereby causing much and offensive effluvia; and even if this be not the cause of the fever, it, at all events, much retards the recovery of those unfortunate patients who live in the vicinity."

*Beccles.*—Mr. Crowfoot says, that scarlet fever, measles, gastric and typhus fever, have been very prevalent; the gastric fever has been accompanied, in many cases, with diphtherite, which has been the occasional cause of death.

*Thetford.*—Mr. Bailey furnishes the following observations for the Quarter:—"September.—The thermometrical range assumed an autumnal one; the difference between the highest and lowest temperature being much less. Both the midnight thermometer and that on the grass were comparatively high. There were only three degrees between the mean temperature and the dew point; this indicated the air to be nearly saturated with moisture, which was the cause of the thick fogs and dew in the mornings. The thermometer was unusually high, being 5° above the average of last September. The highest was 76°, and lowest 50°, the range only 26°. The barometrical mean for the month was 29.88½. Among the most prevailing diseases of the month was diarrhœa, which, in some cases, was so severe as to put on a choleraic form; the cramps in the legs and hands being dreadfully severe, with constant watery evacuations. A sudden and fatal case occurred in a servant girl, who died after two hours, and before any medical aid could be given her. With children and infants the disease was very prevalent, but no death took place. We had also a visitation of measles, imported here by some travellers, whose children were suffering at the time with the disease; the type was mild, requiring but little medical attention, and did not extend beyond a family or two. Two cases of erysipelas came under notice. The first patient was the wife of a labouring man—the face and head became swollen, with high delirium and fever. This lasted some days, and then gradually subsided. Her sister, who came over to nurse her, continued in perfect health during her illness, and having left some days, I was requested to visit her, and found her face affected with severe erysipelatous inflammation; thus showing its infectious nature in this instance. Catarrhs and influenza were less prevalent than usual; but several cases of typhoid fever came under treatment, arising, I consider, more from atmospheric causes than from any predisposition to it from poverty of living, etc. Two or three in a family were attacked. Rheumatism and pleurisy attacked the working labourers, arising from the heat, exposing themselves to sudden changes while under profuse perspiration. The casual diseases under treatment during the month were syphilis, neuroses, nephritis, consumption, gastritis, dyspepsia, and some cutaneous diseases.

"October.—Although we experienced but little wind throughout the month, yet the atmospheric pressure was very variable, the range being 1.2 inch. The temperature was less, the highest degree being 68°, and the lowest, 39°. This unequal pressure caused a higher amount of rain than usual, being 2.5 inches; on the day and night of the 22nd, 1.25 inch fell. The prevalent complaints were chiefly catarrrhal affections, and influenza, attacking elderly persons. Diarrhœa was prevalent in some cases, in a severe form, producing the greatest prostration I ever witnessed. In children it was fatal from exhaustion. Erysipelas occurred in a lady, attacking the face and head, producing considerable constitutional irritation, but which did not extend to any other branch of the family. Several cases of typhoid fever, which may be considered epidemic, attacked families in various parts of the town, running their usual course, and leaving the usual sequelæ, such as anasarcous legs, boils, etc. With children, ulcerated sore throats were prevalent, accompanied with considerable fœtor. One case terminated fatally in a few days. In the neighbourhood, many cases of the same kind occurred, requiring great attention and active treatment. Pleurodynia, rheumatism, and cynanche tonsillaris, were very prevalent during the month, which may be attributed to the variable pressure and moist heat throughout the month. A fatal case of puerperal fever occurred in a lady. Although every medical care and skill were given, she sank, leaving a large family to deplore her loss, and the regret of numerous friends. The casual complaints were, within the month, menorrhagia, tic-douloureux, cancer uteri, gout, indigestion, chicken-pox, and psora.

"November.—We had a high barometrical range during the month, together with a higher temperature than usually occurs at this season; so that the vicissitudes were less felt. A remarkable circumstance was the little wind occurring; especially as the month is generally remarked for high winds and gales. The temperature was 8° higher thaa last November. The diseases were not numerous. By the schedule, it will be observed that some cases of scarlatina are recorded. These cases were confined to only one part of the town; how they were imported, I have been unable to ascertain. The disease has been rather severe; the throat much swollen and inflamed; and the febrile symptoms very high. Every precaution is taken to stop its extension, and at the present time no further case has occurred. Two cases of croup occurred; one terminating fatally and suddenly, and the other, in a girl of ten years, which has yielded to active treatment, and is now convalescent. Catarrhal and bronchial affections are very prevalent. Diarrhœa still continues with us, attacking all grades of society, as in last month. Typhoid fever became epidemic, occurring in isolated places, attacking several in a family at the same time. For many years I have not known the disease so prevalent. Had provisions been dear, the poor would have suffered similar to the prevalence of the disease in 1814. Only one death has occurred. The casual diseases in the month under notice were, inflammation of the colon, abortion, dysphagia, gout, cephalitis, apoplexy, amenorrhœa, syphilis, paraphimosis, porrigo, psora, herpes, and ringworm. Upon inquiry of our veterinary surgeons, I find there has been much disease among the cattle, chiefly lung disease, which has proved very fatal in this neighbourhood. With horses, sore throats, fevers, and bowel disorders have been the prevalent complaints. No disease with the sheep. They were never known more healthy or in better condition."

*Record of Deaths in Twenty-one Parishes (population 9,574) from the Registrar's Book.*

| *Sept.* | | *Oct.* | | *Nov.* | |
|---|---|---|---|---|---|
| Diarrhœa ........ | 2 | Consumption ...... | 2 | Typhoid fever ...... | 1 |
| Choleraic diarrhœa . | 1 | Bronchitis ........ | 2 | Puerperal fever .... | 1 |
| Intermittent fever .. | 1 | Croup ............ | 1 | Bronchitis ........ | 1 |
| Aphtha .......... | 1 | Malig. ulcer of throat | 1 | Croup ............ | 2 |
| Consumption ...... | 4 | Convulsions ...... | 1 | Apoplexy.......... | 1 |
| Asthma .......... | 1 | Congestion of brain. | 1 | Spina bifida ...... | 1 |
| Diseased heart .... | 1 | Spinal disease...... | 1 | Inflamm. of womb.. | 1 |
| Rupture of the aorta | 1 | Atrophy .......... | 2 | Dropsy............ | 1 |
| Apoplexy.......... | 2 | Debility .......... | 3 | Debility .......... | 1 |
| Fits .............. | 1 | Decay of nature .... | 1 | Decay of nature .... | 3 |
| Dropsy............ | 1 | | | | |
| Hanging .......... | 1 | | | | |
| | 17 | | 15 | | 13 |

*Stourbridge.*—Mr. Freer says: "The season has hitherto been dry—the number of chest affections much smaller than usual. Two cases of diphtherite have come under my notice during the last fortnight; one (my nephew), died on the fifth day; the other (sixteen months old), on the fourth. This case of diphtherite occurred near Bewdley, at a country house. The next house is about one hundred and fifty yards distant, and in this a fatal case of the same disease happened about two years ago. There has been a severe epidemic of pleurisy and pneumonia in horses in the neighbourhood, during the last month. I am told depletion is very badly borne in this; many of the animals require ammonia, freely given as a stimulant on account of the early appearance of prostration. Vegetation is forward, as shewn by the blossoming of the strawberry in some instances, and the appearance of the tulip above ground in others."

*Dudley.*—Mr. Houghton says: "377 deaths were registered in Dudley the last quarter, which is far above the average,—175 of these from zymotic diseases. Small-pox and measles have slightly decreased, but there has been a great increase in the number of deaths from diarrhœa and scarlet fever; 99 deaths occurred from diarrhœa; 19 from scarlet fever; 15 from small-pox; 24 from fever; 9 from croup; 3 from hooping cough; 2 from measles; 2 from catarrh; 1 from puerperal fever; and one from dysentery. The deaths from zymotic diseases are one in 2.17 of the whole. Hence the positive and relative mortality of Dudley continues excessive. Three cases of diphtherite have came under my observation. They were well marked. They all occurred in persons in the prime of life and well to do; were all associated with the best sanitary state: all shewed symptoms of alarming prostration, but all recovered. They were treated with tincture of sesquichloride of iron and hydrochloric, and internally with stimulants and nutriments almost *ad libitum.* A strong hydrochloric acid lotion was frequently applied to the exudations with the best result. Influenza was very prevalent amongst cattle and horses."

*Barrowden.*—Mr. Swann says:—"During this quarter measles have appeared at Morcott; and although it has gone through the place, I can hear of no deaths. It has been of a mild character. Diarrhœa has not been so prevalent this quarter; three cases of remittent fever have occurred in Barrowden; but the quarter altogether has been much more healthy than last. The potato disease is very bad, the tubers having gone since they have been housed."

*East Dereham.*—Measles were very prevalent; the disease was generally very mild. Pleuropneumonia had been prevalent among cattle.

*Pontesbury.*—Mr. Eddowes says that one case of small-pox occurred in a girl, aged 16, the daughter of a tramp. She is supposed to have caught the disease at Oswestry.

*Burton.*—Dr. Thomson says: "In my country district we have been remarkably free from epidemic diseases during the quarter; but in the town of Burton I believe that typhus fever, scarlet fever, and small-pox, have been unusually prevalent."

*Oswestry.*—Mr. Cartwright says: "Croup for the most part followed the course of the veins, was severe in character, and bore active depletion. Fever of a remittent type had been general, but not fatal, although of a low character, and requiring early support; delirium, in most cases, has set in as early as the third day. An unusually tardy convalescence has been noticed, characterised by excessive sweating, soft and feeble pulse; puerperal cases were disposed to take the epidemic fever and gave much trouble. During the latter part of the quarter several deaths have taken place in subjects debilitated by irregular living; this has been remarkable."

*Wrexham.*—Dr. Williams writes: "The potato disease has proved most destructive (to that crop) in this neighbourhood. It showed itself earlier than usual; and parties competent to give an opinion, declare that its ravages are more serious than the first year of its appearance. Pleuropneumonia is also very prevalent amongst cattle."

*Alford.*—Dr. West writes: "The cases of cholera were some in which there were profuse rice-water evacuations, with severe cramps, and arrest of the secretion of urine. In one case, in a stout-built man of 70, a single dose of chalk-mixture having completely stopped the diarrhœa, there followed, first, frequent vomitings of dark green matters; then obstinate constipation, which large and frequent doses of castor oil failed to relieve, but which was finally overcome by a repetition of enemata, the obstruction proving to have depended on a collection of *scybala;* the urinary secretion returning at the same time in great abundance, although catheterism was twice necessary to empty the bladder. The man recovered. In another case in a thin spare old man, of about 70, in whom the cramps were especially violent, a single dose of astringent medicine having stopped the diarrhœa (profuse rice-water), there was the same kind of vomiting; complete arrest of urinary secretion, no possibility of restoring the action of the bowels; catheterism, *after forty-eight hours with no water passed,* producing only about half an ounce of urine, and injections bringing nothing away from the bowels. The man died."

*Gainsborough.*—Dr. Mackinder says: "The vicissitudes of the autumnal season, the removal of the oxygen-eliminating foliage, the superabundance of decaying vegetable matter, and the fluctuations of a lower and less genial thermometrical scale, with the concomitant atmospheric phenomena, prepare us for a considerable amount of sickness and mortality, beyond what was recorded in the summer quarter. The increase of deaths has been chiefly at the two extremes of life—the very old and very young: in the former, from paralysis and thoracic affections; in the latter, from dysentery, bronchitis, and the inanition of unnatural or artificial nursing. We have had a few cases of erysipelas, scarlatina, tic, rheumatism, diarrhœa, and dysentery, more of catarrh, and the diseases of the respiratory organs, while the prevalence of ophthalmic and eruptive affections remind us, touchstone-like, of the past visitation of measles. A few cases of fever, some very mild, others of a more aggravated character, have been sprinkled amongst us. Two of these, which assumed the typhoid form, occurred in a clean, well ventilated house, and for some time caused much perplexity as to their origin. At length, while hunting for an imaginary dead rat, the presumed source of an offensive odour, it was discovered that, in erecting a water-closet in an adjoining dwelling, a hole had been made through a wall under the staircase leading to the dormitory of my patients, thus giving exit to the morbific generator of the disease. In one of our narrowest, ill-ventilated, and most offensive yards, we had at one time one case of scarlet and two of maculated fever; all of which recovered despite the absence of a free current of pure air. The only malady entitled to the epithet epidemic, is that unfortunate importation from France, which is exciting so much attention throughout the country—diphtheritis. I have not heard of a death from it, but have seen many cases,—indeed, nearly the whole of my patients are more or less affected by it. In the majority of instances there is no pain experienced until the disease is fully developed, and consequently it has to be sought for in order to treat it in its primary stages. When first observed, the cases might be mistaken for ordinary faucitis and stomatitis, with occasional enlargement of the tonsils; but, on a narrower inspection, it will be perceived that the inflammatory blush is less vivid, more rubeolous and mottled, more diffused, and less abruptly marginated. If the disease be not arrested at this stage, small points of lymph or lymph-like material are deposited on the inflamed membrane. These points then seem to enlarge circumferentially, until, in some cases, the fauces, uvula, and soft palate become enveloped. Small patches are also found on the tongue and about the mouth. In some cases excavation of the mucous surface seems to precede the deposition of the new matter ; in others, I have not observed any such destruction of tissue until the false membrane has been removed; in most cases the integrity of the surface is preserved throughout. Generally there is a very troublesome cough, often gastro-enteric irritation, and nearly always considerable depression, and sometimes absolute prostration of the vital powers. When I could see the case early, I have thought the free application of the solid nitrate of silver mitigated, and, in several cases, arrested the diphtheritic condition, and the chorate of potassa has been my favourite remedy. Whatever may be the true nature of the disease, whether it be an exudation of organisable or inorganisable material, or the deposition of a parasitic cell, which the microscope may hereafter reveal, it is of an adynamic type, and tolerates, if not, demands, the liberal use of stimulants. Ordinary throat affections take on the diphtheritic condition; and the cases of anginose scarlatina which have been recently under my care, have not escaped the epidemic. Endemically we have little to complain of, except it be an apparently incurable disease which has had a chronic existence in the brains of many of the inhabitants of our court-yards, and for the eradication of which our Board of Health will have to tax its ingenuity. It is a sort of *atmospherophobia,*—a horror of fresh air, a dread of draughts. Here, ensconced within the antique appendages of the four posts, like the presiding guardian of an ebon hue, lingers the poisoned expiration of many a distempered form that I visit; whilst, panting without the casement, yearning for admission, pleads despairingly the health-giving air. When formidable malady is annihilated, and a few other matters of minor importance having been attended to, we shall not have occasion, as far as the health of our town is concerned, to quarrel with any one."

The subjoined meteorological report is furnished by Mr. Dyson:—"During the quarter ending November 30th, the weather has been subject to the usual vicissitudes. The month of September was fine and warm, the temperature twice, on the 7th and 17th, having reached 79°. Rain fell on twelve days, and the amount collected was 2.47 inches. The hottest day in October was the 1st, the temperature being 73°. Dense fogs and drizzling rain prevailed from the 12th to the 27th. The amount of rain was 1.7 inch. Dense fogs and mild temperature characterised nearly the whole of November till the 25th, when the temperature suddenly fell in the night to 26°, or 6° below the point of freezing. Rain fell on sixteen days, and the amount 1.12 inch. The total amount of rain in the quarter was 5.39 inches on 45 days. There was a free development of ozone during nearly the whole of September and October; partial in November; on the first four days, and from the 14th to the 22nd. The range of temperature between the 23rd and 25th of November, in 36 hours, was from 52° to 26°, or a difference of 26°. Coughs and sore throats immediately succeeded this sudden change from mild, insinuating, and relaxing weather, to the extreme of winter cold. A severe frost set in on the 28th, but lasted only one day; the wind suddenly changed on the 29th, and the temperature rose 12° in that number of hours."

*Liverpool.*—During the quarter ending on September 20th, 3,428 deaths were registered in the borough, including 189 sudden and violent deaths, and deaths in the hospitals. This number was 410 more than the corresponding quarter of last year, and 70 more than the corrected average of the same quarter of the preceding nine years, excluding epidemic cholera. The deaths from zymotic diseases during the quarter were 1,289, being 115 more than the corrected average without cholera. From diarrhœa the deaths were 618, nearly one-half of the entire number, the average being 405. From hooping-cough the deaths were 190; from scarlatina, 179; fever, 107; small-pox 43 (of which 12 were after vaccination); dysentery, 33; cholera, 33; croup, 16; measles, 16; syphilis, 10. The only diseases of this class more prevalent than usual besides diarrhœa were hooping-cough and scarlatina, the latter of which still showed a disposition to spread. From hooping-cough the deaths during the last five quarters had been successively 55, 65, 100, 158, 190; but the disease appeared to have reached the maximum for the present. The mortality from croup was lower than in any quarter except that immediately preceding; and that of measles lower than in any, with the exception of the quarter immediately preceding. Diseases of the lungs, including 358 from consumption, had caused 644 deaths, being 30 less than the average.

For the deaths in each week, and the meteorological returns, see the opposite table.

*Wigan.*—Mr. Hussey writes: "Catarrh (or influenza) of a mild character generally, has prevailed much since the cold winds set in. Diarrhœa, of a most severe character, with cramps in many instances, has been the order of the past quarter. In

| DEATHS. Week ending | September. | | | | October. | | | | | November. | | | |
|---|---|---|---|---|---|---|---|---|---|---|---|---|---|
| | 5th. | 12th. | 19th. | 26th. | 3rd. | 10th. | 17th. | 24th. | 31st. | 7th. | 14th. | 21st. | 28th. |
| Scarlet fever | 14 | 16 | 15 | 30 | 26 | 21 | 30 | .. | .. | .. | .. | 33 | 25 |
| Measles | 2 | 1 | 1 | 2 | 2 | 2 | 2 | .. | .. | .. | .. | .. | .. |
| Small-pox | 1 | 4 | 3 | 5 | 3 | 2 | 2 | .. | .. | .. | .. | .. | 4 |
| Hooping cough | 16 | 12 | 8 | 15 | 12 | 21 | 15 | .. | .. | .. | .. | .. | 11 |
| Croup | .. | .. | 1 | 2 | 1 | 2 | .. | .. | .. | .. | .. | .. | 2 |
| Cholera | 2 | 1 | 3 | 36 | 24 | .. | 1 | .. | .. | .. | .. | .. | .. |
| Diarrhœa* | 73 | 50 | 47 | 36 | 3 | 20 | 13 | .. | .. | .. | .. | .. | 2 |
| Typhus | 14 | 9 | 12 | 13 | 14 | 17 | 10 | .. | .. | .. | .. | .. | 17 |
| Disease of lungs | 34 | 45 | 39 | 47 | 44 | 62 | 59 | .. | .. | .. | .. | 100 | 103 |
| Purpura | .. | .. | 1 | .. | .. | .. | 3 | .. | .. | .. | .. | .. | .. |
| Intemperance | .. | 1 | 2 | .. | .. | .. | .. | .. | .. | .. | .. | .. | 1 |
| Syphilis | 2 | .. | 1 | 1 | .. | 1 | .. | .. | .. | .. | .. | .. | .. |
| Total deaths during each week | 272 | 272 | 237 | 250 | 232 | 235 | 254 | 278 | 261 | 316 | 352 | .. | 266 |
| Corresponding week, 1856 | .. | .. | .. | 240 | 192 | 185 | 203 | .. | .. | .. | .. | 192 | 209 |
| **METEOROLOGICAL OBSERVATIONS.** | | | | | | | | | | | | | |
| Barometer: Mean | 29·79 | 29·69 | 30·19 | 30·10 | 29·98 | 29·50 | 30·03 | 29·90 | 29·88 | 29·92 | 30·54 | 30·28 | .. |
| Dew-point temperature | 54·6 | 56·5 | 59·5 | 51·9 | 53·5 | 47·3 | 54·1 | 47·5 | 47·8 | 48·4 | 45·5 | 44·4 | .. |
| Thermometer: Highest (mean) | 67·1 | 65·5 | 69·4 | 64·1 | 64·3 | 57·7 | 62·2 | 58·3 | 57·0 | 54·8 | 52·0 | 53·5 | .. |
| Lowest (mean) | 55·7 | 56·5 | 58·3 | 53·5 | 55·5 | 48·9 | 53·5 | 49·6 | 48·6 | 47·7 | 44·5 | 46·2 | .. |
| Mean | 61·4 | 61·0 | 63·8 | 58·8 | 59·9 | 53·3 | 57·8 | 54·0 | 52·8 | 51·3 | 48·3 | 49·8 | .. |
| Mean of 11 years | 60 3 | 58 8 | 57·5 | 57·0 | 55·1 | 53·1 | 51·4 | 51·7 | 49·0 | 49·0 | 46·6 | 44·9 | .. |
| Range | 11·4 | 9·0 | 11·2 | 10·5 | 8·7 | 8.9 | 8·7 | 8·8 | 8·4 | 7·1 | 7·5 | 7·4 | .. |
| Mean range, 11 years | 10·3 | 10·9 | 10·1 | 9·3 | 8·6 | 8·1 | 8·4 | 7 6 | 7·7 | 7·2 | 6·8 | 6·4 | .. |
| Rain fell on days | 4 | 3 | 1 | 2 | 3 | 6 | 1 | 4 | 3 | 4 | 0 | 2 | .. |
| Rain fell during hours | 11·7 | 1 | 2 | 1·9 | 12 | 16·7 | 1·4 | 6 | 7·5 | 27·2 | 0 | 7 | .. |
| Amount of rain in inches | 0·71 | 1·39 | 0·04 | 0·08 | 0·80 | 1·27 | 0·02 | 0·32 | 0·24 | 0·81 | 0 | 0·26 | .. |
| General direction of wind | N. | var. | SE. | SE. | SW. | var. | S. | N. E. | SE. | SE. E. | S. SE. | S. SE. | .. |

* Of the 73 deaths from diarrhœa, noted on September 5th, 60 were in infants. Of the 50 deaths, noted September 12th, 44 were in children; two only in persons between 4 and 76 years; and four in men aged respectively 76, 83, 92, and 97. Of the 47, noted September 19th, 42 were in children under 2 years of age. Of the 36, noted September 26th, 34 were in children.

Wigan, I have been informed by Mr. Winnard, smallpox has been very rife, and what is remarkable is, that in the *confluent form the patients have gone about the house comparatively free from constitutional symptoms or complications.* In badly drained districts typhus seems to linger on, but typhoid fever has been more general. Children of the poor and middle classes have of late suffered much from bronchitis with diarrhœa, and, in a few cases, mild dysentery. In my pauper practice, I have no epidemic cases, notwithstanding the cold weather and bad times, and in the workhouse we have nothing of any note—nor for three months past. The smallpox in Wigan has prevailed much amongst adults, a fact which in England calls loudly for revaccination, as is the practice in America. November was our coldest month; there was little or no rain."

*Bolton-le-Moors.*—Mr. Pendlebury says: "There has been a considerable amount of sickness during the past quarter; in the latter half of which there has arisen a rather fierce epidemic of influenza. Bronchial affections have been very prevalent, especially in children, amongst whom there was scarcely ever known to have been a greater mortality. Several cases of the disease, lately noticed in the county of Essex, have been observed, but of a milder form. At the onset, three cases resembled ordinary quinsy, accompanied with great debility, lassitude, and general *malaise;* but the tonsils (on the second or third day), instead of suppuration, exhibited superficial brown sloughy patches. The lunar caustic, with mineral acids and quinine, generally effected a cure. Typhus has not so generally prevailed in the town as in the country. A few cases only of small-pox have appeared. The other exanthemata have been ordinarily rife. The autumnal season was never known to have been finer, or more suitable for agricultural purposes. The winds have almost constantly held to the E. and S.E., with an unusually small quantity of rain. Cases of rheumatism, and especially of the acute variety, have been quite the exception this quarter. I have been informed that, in some of the colliery districts, wounds from accidents have had a great tendency to an unhealthy action; many cases simulating hospital gangrene."

*Bramley*—Mr. Radcliffe says: "Epidemics, catarrh (coryza) were prevalent during the weeks ending Nov. 6th, 13th, 20th, and 27th, but the cases were not of sufficient severity to require medical interference. Well marked cases of influenza occurred during the weeks ending November 20th and 27th (during the same period influenza was very prevalent in the neighbouring town of Leeds); common continued fever was rife during the weeks ending 13th, 20th, and 27th. The majority of the cases of fever were complicated with bronchitis. No case of epizootic pleuropneumonia among horned cattle, or of any other epizootic affection, has come to my knowledge in the township during the past quarter. From one-third to two-thirds of the different crops of the potatoes in the township have been destroyed by the potato-disease."

*York.*—Mr. Procter writes: "October and November were in this place generally unhealthy, but the diseases were not fatal except in the aged. Small-pox prevails extensively. The cases of scarlatina have been numerous, not fatal, but usually with more or less throat affection and a prolonged convalescence. The influenza in its very general, almost, it may be said, universal attacks, has been very frequently accompanied by a low typhoid fever and in some cases by pneumonia."

*Rothbury.*—Mr. Summers says: "The cases of typhus were not numerous, but some of them were very severe. Two fatal cases were children of ten or twelve years of age, both residing in places surrounded by filth, &c. In one case an open drain runs close along the back wall of the house, receiving the filth of several houses, and of a butcher's slaughter-house. The house is besides surrounded by dungheaps and stables. In the other case, there existed a choked up drain on the premises, to the danger of which the tenant's attention had been drawn only a short time before the occurrence of the fever."

*Lerwick.*—Dr. Spence says: "The fine weather mentioned as prevailing during last quarter continued during September and October, these months being finer and more equable than usual: but a sudden change took place about the beginning of November, when the weather became cold, wet, and stormy. The indications of ozone were greatest in the beginning of September and the end of November, and least in the middle of October. It appeared to me that when ozone was deficient diarrhœa was common, and when it was above the average, catarrhs were prevalent. Influenza was pretty common in the last week of the quarter, during which the indications of ozone were higher than at any other period since I commenced my observations. Diarrhœa was of much more frequent occurrence than in ordinary seasons; many of the cases, especially during the week ending 16th October, very severe, and attended with vomiting and cramps. I have separated the cases of common continued fever and typhoid fever, or dothinenterite, from typhus, to avoid what I look on as a grave error in epidemiology. The case of typhoid occurred in a young man who had returned from the Davis's Straits whale-fishery by way of Peterhead, and who was feeling unwell when he landed in Lerwick. The total number of deaths from all causes was eighteen, of which eight occurred in November; the average age was forty-seven, there being three below five, and five above seventy. The causes in those cases in which medical certificates were furnished were, dysentery, one; typhoid fever, one; pneumonia, one; cerebral disease, one; acute hydrocephalus, one; infantile convulsions, one; cardiac disease, two; chronic bronchitis, one; cancer of lung, one; chronic hepatitis, one; malignant ulcer of leg, one; and irritative fever caused by the prick of a rusty nail, one."

